# Spatial Differentiation of Digital Rural Development and Influencing Factors in the Yellow River Basin, China

**DOI:** 10.3390/ijerph192316111

**Published:** 2022-12-01

**Authors:** Jiamin Ren, Chenrouyu Zheng, Fuyou Guo, Hongbo Zhao, Shuang Ma, Yu Cheng

**Affiliations:** 1 College of Geography and Environment, Shandong Normal University, Jinan 250358, China; 2 Northeast Institute of Geography and Agroecology, Chinese Academy of Sciences, Changchun 130012, China; 3 College of Resources and Environment, University of Chinese Academy of Sciences, Beijing 100049, China; 4 School of Geography and Tourism, Qufu Normal University, Rizhao 276826, China; 5 Key Research Institute of Yellow River Civilization and Sustainable Development & Collaborative Innovation, Center on Yellow River Civilization Jointly Built by Henan Province and Ministry of Education, Henan University, Kaifeng 475001, China; 6 Institute of Agricultural Information and Economics, Shandong Academy of Agricultural Sciences, Jinan 250100, China

**Keywords:** digital rural, spatial heterogeneity, geodetector, influencing factors, Yellow River Basin

## Abstract

The new development mode represented by the digital economy has provided new ideas for sustainable rural development. To comprehensively understand the status of digital rural development and propose scientific measures of rural revitalization in the Yellow River Basin (YRB), this study used counties as the research unit and data from 2020 to analyze the spatial differentiation characteristics and influencing factors by employing the Theil index, spatial autocorrelation analysis, and a geodetector model. The results showed that the digital rural development index in the YRB is slightly higher than it is in China overall, but the sub-index for the digital economy is lagging. The levels of digital rural development in the different reaches were lower reaches > middle reaches > upper reaches. Additionally, municipal districts and county-level cities have higher statuses than t general counties. Moreover, the decomposition of the Theil index shows that the intra-group differences in the upper reaches and general counties are the most important cause of the total differences. Moreover, the levels of digital rural development demonstrate spatial differences, with high and low levels in the east and west, respectively. An obvious reliable spatial correlation exists, and the spatial agglomeration featured with a similar level is significant. Finally, the influencing factors of spatial heterogeneity of digital rural development in the YRB and different reaches were different, with government expenditure being the main leading factor in the YRB and its upper reaches, while educational attainment and industrial structure are the leading factors in the middle reaches and lower reaches, respectively. The explanatory power of the interactions between the factors far exceeds that of a single factor, as shown through double-factor and nonlinear enhancement. This study provides a scientific reference for facilitating more targeted policy measures to achieving the goal of digital China and rural revitalization.

## 1. Introduction

Sustainable development in rural areas has always been at the heart of regional development. In 2015, the United Nations (UN) proposed 17 Sustainable Development Goals to be achieved according to a 15-year plan. Goal 9.3c aims to “Significantly increase access to information and communications technology and strive to provide universal and affordable access to the Internet in least developed countries by 2020.” (https://www.un.org/sustainabledevelopment/development-agenda, (accessed on 8 July 2022). More rural areas are becoming connected to urban hubs and global markets via information and communications technology (ICT) [1]. Despite digital connectivity, geographic and social inequalities, such as the digital divide and economic disempowerment, persist in rural areas [2,3]. These problems raise the question of whether the digital divide can be eliminated by a series of concessions. Digital transformation in rural areas is a policy of priority around the world. For example, the European Union Commission has put forward a project that aims at “fully connecting farmers and countryside to the digital economy” [4].

China is the largest developing country in the world and has a large agricultural sector, which was dominant for a long time. The rural population constitutes the majority of the country’s population and is distributed over a very wide area [5]. Rural development has always been the primary focus of the central government. Since China’s economic reforms and liberalization, its rural environment has experienced numerous changes, with the emphasis shifting from agricultural production, social stability, cultural heritage, and ecological conservation [6]. Numerous problems, such as hollow villages, poor counties, population aging, and environmental deterioration, have appeared [7,8,9]. The central government has implemented a range of policies to promote the development of the rural areas. In 2017, General Secretary Xi Jinping proposed the Rural Revitalization Strategy (http://www.gov.cn/zhuanti/2017-10/27/content_5234876.htm, (accessed on 8 July 2022). With the rise of the digital economy, digital rural development has become an important part of this strategy. In 2019, the State Council issued the Digital Rural Development Strategy Outline, which singled out 117 counties and municipalities as testbeds. In 2022, the Digital Rural Development Action Plans for 2022–2025 outlined the objectives, main tasks, and measures. Consequently, digital rural development has become an important issue in China’s overall rural development and revitalization.

Digitalization is a term used to describe the sociotechnical processes surrounding the use of digital technologies that affect social and institutional contexts [10]. The State Council [11] views digital rural development as an endogenous process of agricultural and rural modernization and transformation via the application of networking, informatization, and digitalization in the economic and social development of agricultural and rural areas as well as the improvement of the modern information skills of farmers. Rijswijk et al. [12] proposed digital transformation as systemic changes that affect how people, things, and institutions coordinate themselves to perform their activities. Wang et al. [13] stated that digital rural development was a means, process, and state of reconstructing rural economies by relying on the development of the digital economy. In conclusion, digital rural development involves the transformation of production, lifestyle, and governance modes. Measurements of such development have suffered from a lack of data about digital finance [14], digital poverty [15], and villages specializing in e-commerce [16]. In addition, policies and measures are also key areas for academic fields [17,18]. In terms of literature review, this paper intends to fill the research gaps from the following perspectives: First, limited by the previous data acquisition methods, related research mostly focuses on qualitative discussions or quantitative research based on a few indicators. Second, the influencing factors need to be further analyzed via an evaluation model that considers the spatial heterogeneity, which is being applied less in this field. Moreover, the applicability of these studies on river basins with huge natural and economic differences needs to be explored further. 

The Yellow River Basin (YRB) is the second-largest river basin in China and accounts for 8.3% of the country’s land area. The annual runoff accounts for only 2% of the country’s total annual runoff but supports 12% of the country’s population and 15% of the arable land [19]. A densely populated agricultural area, YRB is significant in the fight against poverty. Recently, concentration of specialized villages has been increasing. The prominent human-land contradiction in the YRB has resulted in increasing numbers of desolate villages with no skilled labor, low income, plentiful idle homesteads, and loose administrative organizations [20,21]. Therefore, to provide a scientific basis for constructing a digital China at the county-level and rural revitalization and to formulate reasonable and effective polices, we took the YRB as the study area, and the Theil index, spatial autocorrelation, and geodetector model was used to investigate the spatial patterns, different characteristics, and driving factors of digital rural development at a county scale. The main objectives of this research are as follows: (1) identify the status and spatial patterns of digital rural development in the YRB; (2) reveal the differentiation and spatial heterogeneity of digital rural development; and (3) explore the factors affecting this spatial variation and provide a reference for follow-up studies.

## 2. Materials and Methods

### 2.1. Study Area

**Figure 1:**
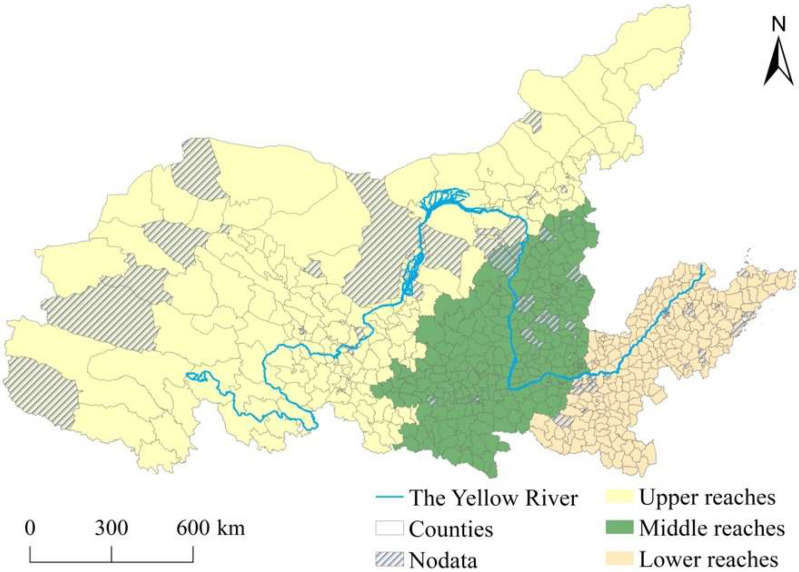
Sketch map showing in the study area.

The YRB originates from the Qinghai-Tibetan Plateau and pours into the Bohai Sea. It flows through the nine provinces and autonomous regions of Qinghai, Sichuan, Gansu, Ningxia, Inner Mongolia, Shaanxi, Shanxi, Henan, and Shandong. However, Sichuan Province is included in the Yangtze River Economic Belt, while the eastern part of the Inner Mongolia area is located closer to Northeastern China. The scope of the YRB contains Qinghai, Gansu, Ningxia, Inner Mongolia (excluding the eastern area), Shaanxi, Shanxi, Henan, and Shandong (Figure 1) [22]. With the exception of counties or county-level cities, this study considered municipal districts in which agriculture accounted for more than 3% of the GDP in 2019. Finally, 648 county-level administrative districts were selected as the study area, of which 28 counties had been listed as experimental sites for digital rural development in 2020 (Details are shown in Table A1, Appendix A).

### 2.2. Data Sources

The 2020 index for digital rural development in each relevant county was obtained from the Institute for New Rural Development, Peking University [23]. The index is derived from the statistical results of the full sample data obtained by Umeng+, the Alibaba group (taobao, Tmall, Alipay, Ali cloud, and other core platforms), Amap, etc., and was developed by New Rural Development, Peking University and Aliresearch. The calculation process includes empowerment, standardization, etc. The index is made up of four parts: digital infrastructure, digital economy, digital governance, and digital life. Compared to the 2018 index [24], it added four basic indicators that considered logistics networks, data platforms, and digital governance tools, meaning that the index can determine the overall trends, main weaknesses, and development potential of China’s digital rural development at the present stage more comprehensively.

The socioeconomic data were mainly obtained from the China County Statistical Yearbook 2020 [25] and from the 2020 China Population Census by County [26]. Some missing data were supplemented using municipal statistical yearbooks and statistical bulletins. The road network data were obtained from OpenStreetMap (www.openstreetmap.org) (accessed on 12 August 2022).

### 2.3. Research Methods

#### 2.3.1. The Theil index

Proposed by Henri Theil in 1967, the Theil index was originally used for quantitative analyses of income imbalances and regional differences. It proved to be superior to other inequality measures, such as mean deviations and the Gini index, because it allows inequality to be divided into within- and between-group components [27]. The counties in the YRB were divided into different groups according to their regions and administrative levels; then, the differences within each group were calculated using the Theil index. As per Li et al. [28], the decomposition of the Theil index can be expressed as:

(1) $$T=\frac{1}{n}\sum_{i=1}^{n}\frac{y_{i}}{\bar{y}}\log\left(\frac{y_{i}}{\bar{y}}\right)$$

(2) $$T=T_{b}+T_{w}=y_{k}\log\frac{y_{k}}{n_{k}/n}+\sum_{k=1}^{k}(\sum_{i\in g_{k}}\frac{y_{i}}{y_{k}}\log\frac{y_{i}/y_{k}}{1/n_{k}})$$

where *T* is the Theil index, *T_b_* and *T_w_* are the differences between and within groups, respectively. *K* represents the number of groups; *y_i_* and *y_k_* represents the levels of digital rural development in unit *i* and group *k*, respectively, and $\bar{y}$ represent the average level.

#### 2.3.2. Spatial Autocorrelation Analysis

Global and local spatial autocorrelation analyses can find the degrees of similarity between the attribute values of a certain spatial region and its neighboring regions [29,30]. We used global Moran’s I to evaluate the global spatial correlation of digital rural development in the YRB:

(3) $$I=\frac{n}{S_{0}}\frac{\sum_{i=1}^{n}\sum_{j=1}^{n}w_{ij}Z_{i}Z_{j}}{\sum_{i=1}^{n}Z_{i}^{2}}$$

where, *Z_i_* is the is the deviation of an attribute for region *i* from its mean, *w_ij_* is the spatial weight between regions *i* and *j*, *n* is equal to the total number of features, and *S*_0_ is the aggregate of all of the spatial weights. The queen contiguity weight matrix was used here. The *Z*-score and *p*-value are measures of statistical significance, so if either shows statistical significance, then a positive or negative Moran’s I index value would indicate a tendency toward clustering or dispersion, respectively.

Global spatial autocorrelation cannot reflect the specific spatial location of clustering, so a local Moran’s I [31] is used to detect the spatial clustering conditions of digital rural development:

(4) $$I_{i}=\frac{x_{i}-\bar{X}}{s_{i}^{2}}\sum_{j=1,j\neq i}^{n}w_{ij}\left(x_{j}-\bar{X}\right)$$

(5) $$S_{i}^{2}=\frac{\sum_{j=1,j\neq i}^{n}w_{ij}{\left(x_{j}-\bar{X}\right)}^{2}}{n-1}$$

where *n* equates to the total number of features, while the other variables are the same as in Equation (3). According to the *Z*-score or *p*-value, the spatial clusters can be classified into high—high, low—low, high—low, low—high, or not significant.

#### 2.3.3. Construction of Influencing Factor Model

(1) Selection of Explanatory Variables for Digital Rural Development

Digital rural development is multidimensional, so its influencing factors need to be considered from many angles. Based on digital technology and characterized by the digital economy, digital rural development can remove dependence on the natural geographical environment to the greatest extent possible. On the basis of the relevant references and considering the feasibility of the quantification of influencing factors and the availability of data, this paper selected nine factors considering economic basis and the potential of digital rural development (Table 1). It is important to point out that the explanatory variables are not included in the 2020 index evaluation system.

The economic basis can provide a more favorable environment for digital rural development, which cannot exist without a real economy. In general, counties with high economic development can be a positive force for digital rural development, so we selected per capita GDP (X1). Digital rural development also has an important connection with regional industrial structure [32], for which we selected the proportion of the secondary and tertiary industries’ output value (X2) here. A high degree of consumer market activity can provide better access to the digital economy, so we selected the retail sales of consumer goods per capita (X3) [33]. Transportation infrastructure is an important support for the development of rural e-commerce. Accelerating the development of logistics and express delivery can reduce the transaction time of rural e-commerce products [34], so we selected the density of railways and highways (X4) to reflect the transportation infrastructure. 

Relevant studies have found an obvious digital divide between urban and rural areas regarding the development of the Internet and related industries [1], so the urbanization level (X5) has been included in the evaluation system of influencing factors determining the differences in digital rural development. The per capita disposable income of households (X6) in a region was chosen to represent purchasing power [33]. The potential of digital rural development cannot be separated from the government support [35]. Since the government’s decisions are difficult to quantify, we selected the general public budget expenditures per capita (X7) to represent this support. Moreover, the combination of basic knowledge and literacy of digital technology determine the ability of residents to integrate into the digital era. Generally speaking, the higher the education level and the stronger the cognitive ability of the population, the more obvious the promoting effect on digital rural development is. Average educational attainment (X8) was chosen to represent this. In addition, the limited usage capacity for ICT tools makes it difficult for elderly people to adapt the life style prevalent in the digital age [36]. Here, we selected the proportion of working-age people (X9) to reflect the population structure.

(2) Geodetector Model

Geodetector is a new statistical tool that can be used to detect Spatial Stratified Heterogeneity (SSH) and reveal the driving force of geographical objects [37]. This method can test the coupling between two variables, Y and X, according to their SSHs without assuming that the association is linear and can be used to investigate the interaction between two explanatory variables, X1 and X2, in response variable Y without any specific form of interaction, such as the assumed product in econometrics [38]. It has already been applied in many fields of nature and social science [39,40]. It consists of four models: factor detector, risk detector, interaction detector, and ecological detector. In this paper, the degree of influence of different factors on digital rural development is analyzed using the factor detector and interaction detector. The equation is as follows:

(6) $$q=1\;-\frac{\sum_{h=1}^{L}N_{h}\sigma_{h}^{2}}{N\sigma^{2}}$$

where *N* and *σ*^2^ are the number of units and the variance of Y in a study area, respectively. H = 1, …, *L* represent the strata of indicators. Where *q* is the degree of the explanatory power of the influencing factors for digital rural development, its value is strictly within [0, 1].

The closer *q* is to 1, the higher the degree of influence on digital rural development. The discretization of independent variables is the key to detect influencing factors; here, independent variables were discretized by the nature breaks (Jenks) method. Meanwhile, we used the geometrical interval method in ArcGIS and the K-means method in SPSS for robustness analysis. The results suggest that when the independent variables were stratified to strata 7, the q value had no pronounced changes, which indicates that our classification is reasonable.

## 3. Results

### 3.1. Overall Characteristics of Digital Rural Development in the YRB

#### 3.1.1. Comparison of Digital Rural Development in the YRB and China

Table 2 shows the descriptive statistical analysis of the digital rural development indexes and sub-indexes for China overall and the YRB. The mean value of the index for the YRB (56.736) is slightly higher than the overall value (55.73). In the YRB, Boxing County in Shandong Province has the highest score (94.94) and ranks 27th in China, whereas Tongde in Qinghai Province has the lowest score (20.00) and also ranks the lowest. The mean values of the sub-indexes of digital infrastructure, digital governance, and digital life in the YRB are also higher than the overall value. The status of digital governance in the YRB is better than the overall level of digital governance. However, the average value of the digital economy in the YRB lags behind its value overall. Mudan District in Heze City has the highest score (111.38), but a significant gap exists between it and Deqing County (154.87) in Zhejiang Province. The standard deviations and coefficients show the differences among the counties in the YRB to be smaller than those overall. The intercounty gap is mainly observed in the digital economy, but there are less differences in digital infrastructure, thus indicating that the current public infrastructure, as represented by mobile communications networks, is well developed and has become an important foundation for digital rural development.

#### 3.1.2. Internal Differences in Digital Rural Development of the YRB

Table 3 shows the scores of each index divided by location and type of administrative region. The level of digital rural development in the lower reaches is the highest, followed by the middle and upper reaches. The four sub-indexes of digital infrastructure and digital economy also have the same ranking. In addition, the average scores of the counties located in the upper reaches are much lower than those in the other reaches, especially for digital infrastructure, which has a 17.968 difference from the average.

The digital rural development of municipal districts and county-level cities is better than that of general counties, whose scores are lower than the average level. Marked differences exist between the sub-indicators for the digital economy and the infrastructure of the ordinary counties and municipal districts (county-level cities), but relatively small differences exist for digital governance and digital life. Currently, the 28 national experimental sites for digital rural development were higher than the average level, especially for digital economy and digital life, while there were still gaps in digital infrastructure and digital life compared to the whole YRB.

Table 4 shows the decomposition results of the Theil index of digital rural development by region and the administrative level of each county (district).

According to the location of each county (district) in the basin, the intra-regional differences accounted for 57.49% of the overall difference in 2020, indicating that the intra-regional difference was the main reason for the differences in digital rural development, but the influence of inter-regional differences on the overall difference should not be ignored. Specifically, within each group, the differences within each region in 2020 are ranked according to the upper, middle, and lower reaches, with little difference in the proportion of differences between the upper and middle reaches but with the smallest proportion of differences in the lower reaches. In general, the differences in digital rural development among counties in the upper and middle reaches are the most important factor causing intra-group differences. Regarding administrative attributes, the differences within each group accounted for 89.38% in 2020, which was much higher than the differences between groups (10.62%). The results show that the differences within each group were the main factor causing the overall difference, whereas the differences between groups was a secondary factor. Specifically, the proportion of differences among ordinary counties within each group was much higher than that of municipal districts (county-level cities), so the differences within the group of ordinary counties are an important reason for the overall differences.

### 3.2. Spatial Patterns of Digital Rural Development in the YRB

#### 3.2.1. Spatial Distribution Pattern

**Figure 2:**
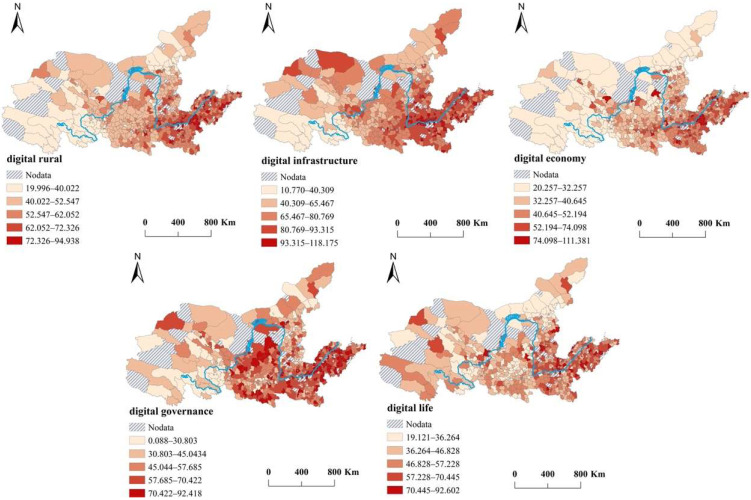
The spatial patterns of digital rural development in the YRB, 2020.

We used the natural breaks method to classify the digital rural development indexes and four sub-indexes of the 648 county-level units. The results were visualized by ArcGIS (Figure 2). The spatial differentiation of digital rural development is significant because it shows a relatively typical watershed distribution. Counties, such as Qingdao in the Shandong Peninsula, Heze in southwest Shandong, and the surrounding areas of Zhengzhou in Henan Province, which all have high levels of digital rural development, are mainly concentrated in the lower reaches. A total of 63 counties (cities) belong to this type and account for 9.72% in the study area. In contrast, counties, such as Qinghai Province, the north of Gansu Province, and the central part of the Inner Mongolia Autonomous Region, which have low levels of digital development, are mainly distributed in the upper reaches and form large spatial agglomerations. The number of counties in this type of area accounts for 8.49% of the total counties. The number of counties with intermediate levels of development is significantly higher than the number of counties for the other levels and accounts for 28.36%. Their spatial distributions are also significantly different, with wide dispersions and small agglomerations.

The spatial distribution pattern of the digital economy index is similar to that of the digital rural index, indicating that the digital economy is an important factor affecting the construction of the digital rural. For the other three sub-indexes, the number of counties with low scores for digital infrastructure is small, their spatial distribution scope has been significantly reduced, and most of them are distributed in Qinghai Province. These results are mainly due to the relatively poor location conditions of these counties, so their levels of economic development require improvement, which creats difficulties for the promotion of digital infrastructure. The regions with good digital infrastructure development are mainly located in Henan and Shandong Provinces in the middle and lower reaches, which are also the regions with high economic development and population density. The spatial distribution of the digital governance index is relatively uniform, as even remote areas in the upper reaches have counties with high scores. This distribution also reflects the efforts of local governments to become more service-oriented in the new era. For the digital life index, which is significantly different from the low value of the digital rural development index, the low-value areas are mainly distributed in the Inner Mongolia Autonomous Region.

#### 3.2.2. Spatial Agglomeration Characteristics

GeoDa is a free and open source software tool was developed by Dr. Luc Anselin and his team. The spatial autocorrelation statistics module of GeoDa 1.18 were used to calculate the global Moran’s I index of digital rural development (Table 5). 

The values of Moran’s I for digital rural development and those of the other four sub-indexes were greater than 0 at a significance level of 0.001, indicating that digital rural development has a spatial agglomeration trend. Digital infrastructure has the strongest spatial agglomeration trend, followed by the digital economy and digital management, whereas digital life index has the weakest degree of agglomeration. 

**Figure 3:**
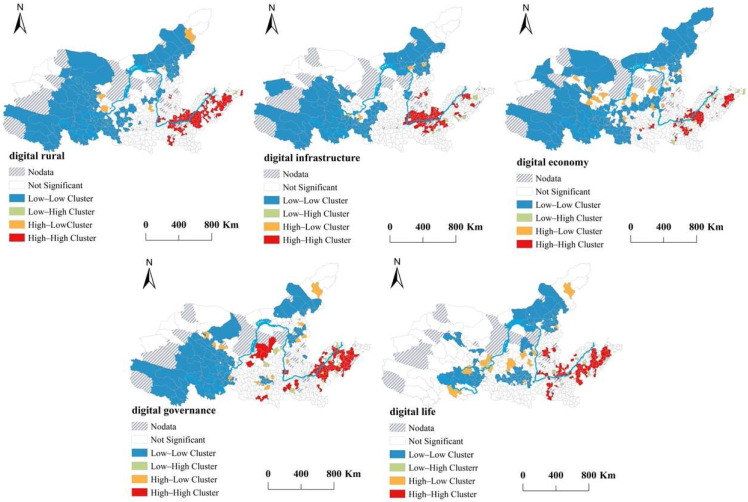
The LISA distribution of digital rural development in the YRB, 2020.

The local Moran’s I index was used to further analyze the local spatial features of digital rural development in the YRB (Figure 3). The digital rural development index forms two obvious high—high agglomerations in the lower reaches of the Shandong Peninsula and around Heze, Kaifeng, Zhengzhou, and other cities at the borders of Henan and Shandong Provinces, whereas the low—low agglomerations are mainly distributed in Qinghai, Gansu, Inner Mongolia, and the eastern part of Gansu Province.

According to the sub-index for digital infrastructure, the low—low clusters are generally similar to those mentioned above. The high—high clusters are mainly distributed in the southeastern counties of Shaanxi Province and the northern counties of Henan Province, as well as in some counties, such as Dongying, in the Yellow River Delta of Shandong Province. 

In comparison, the spatial distribution range of the low—low clusters of the digital economy index is the widest, while the spatial range of the high—high cluster is significantly reduced and is only distributed in Qingdao, Heze, and Shandong Provinces, as well as in the areas surrounding Luohe City, Henan Province. According to the digital governance index, the counties in northwest Shaanxi Province, in addition to those in Shandong Province, also present a spatial distribution of high—high agglomeration. The low—low agglomerations are also distributed in the northern counties of Shanxi Province in addition to in Qinghai. For the digital life index, the high—high agglomerations are mainly distributed in the Shandong Peninsula and in some counties in southern Shandong Province, while a small part is distributed around central cities such as Zhengzhou City in Henan Province and Taiyuan City in Shanxi Province. Compared with the other indexes, the distribution range of the low—low agglomeration is significantly reduced, forming three small areas of low—low agglomerations. In general, the counties with high levels of digital rural development are mainly distributed in the central cities of the Shandong Peninsula and around cities such as Zhengzhou in Henan Province, while the cities with low levels digital rural development are mainly distributed in the upper reaches.

### 3.3. Influencing Factors of Digital Rural Development

#### 3.3.1. Driving Factor Detection

As shown in Table 6, all detection factors passed the significance inspection, indicating that the selected driving factors could better explain the spatial differentiation patterns of digital rural development. 

The top five factors affecting digital rural development in the whole YRB were X7 > X4 > X3 > X8 > X2. This indicates that the government support at the present stage has played a leading role in digital rural development. The results of the interaction detection also confirm this conclusion. Transportation infrastructure is another important influencing factor, and it has been shown that traffic conditions are an important support for the development of rural e-commerce. Accelerating the development of logistics and express delivery on this basis can reduce the transaction time of rural e-commerce products and improve the competitiveness of rural commodities. In contrast, the effect of the per capita GDP (X1) and the proportion of working-age people (X8) is weak.

The results of factor detection show that the driving factors of digital rural development in the different reaches have obvious differences. Many factors (except for X6 and X9, other factors have passed the significance test) have influenced digital rural development in the counties of the upper reaches, showing obvious hierarchical differences. Government expenditures are the key factor leading the development of digital rural development in the upper reaches. Road network density (0.356), average educational attainment (0.275), and industrial structure (0.252) are the second group of leading factors affecting digital rural development in the upper reaches, in which rational layout, the optimization of the industrial structure, the coordinated development of the secondary and tertiary industries, and improvements in basic implementation and construction are effective ways to promote digital rural development. In comparison, the explanatory powers of market size (0.177), urbanization level (0.148), and economic development (0.050) are relatively weak.

For the counties in the middle reaches, average educational attainment (0.451) plays a significant role in the development of digital rural developement. The influences of development potential factors on the differences in their development are stronger than those of economic basis factors, indicating that digital rural development has mainly been affected mainly by the factors of development potential factors. Compared with the upper reaches, t road network density did not pass the significance test. 

The influencing factors of digital rural development in the lower reaches have mainly been affected mainly by the industrial structure (0.150), which is the dominant factor, and the level of economic development (0.126). The other factors did not pass the significance test. As the region with better economic and social development, the lower reaches have gradually reduced their reliance on the government to promote the construction of the digital rural, which has been more dependent on endogenous factors. Some counties are still major grain producers. In the future, agricultural and sideline product processing as well as other industries, could be further developed on the basis of the existing industry, and in-depth digital integration could be conducted to develop e-commerce and other new industries for the development of the rural digital economy.

#### 3.3.2. Results of Interaction Detection

The effects of interactions among driving factors on the spatial patterns of digital rural development in 2020 were detected by the interaction model known as geodetector. Table 7 presents the main top-ranking interaction effect.

When any two independent variables act on digital rural development at the same time, the effects of interactions between different factors are enhanced by double-factor and nonlinear enhancement in the whole YRB, more than a single variable, and no weakening or independent-type of effect is observed. This indicates that the spatial pattern was the result of the combined action of the driving factors. The interaction between government financial expenditure and other factors is the strongest, with explanatory power of more than 57.9%, followed by the interaction between road network density and other factors, with explanatory power more than 41.7%, indicating that the development of the spatial pattern of digital rural development is mainly influenced by government support, traffic accessibility, and other factors. In addition, X1 in factor detection has a relatively weak influence (0.081); however, when X1 interacts with other variables, the q value has a remarkable increase, indicating that the effect of X1 on digital rural is indirect and that it drives the construction of industry, infrastructure, education, market, etc., indicating that that it can significantly promote the development of digital rural.

Concurrently, the interactions between any two driving factors were different in different reaches. For the upper reaches, X4 ∩ X8, X4 ∩ X5, X7 ∩ X8, and X4 ∩ X9 had the most significant effects on digital rural development; traffic conditions became the main interacting factors; and the interaction of education level became important. X7 ∩ X8, X8 ∩ X6, X8 ∩ X9, and X5 ∩ X7 had the most significant effects on the digital rural development in the middle reaches, and the interaction between government financial expenditure and other factors was the strongest. Since most variables in the lower reaches did not pass the significant test, only the interactive detection results between X1 and X2 showed a nonlinear enhancement trend.

## 4. Discussion

### 4.1. Spatial Pattern of Digital Rural Development in the YRB

The emerging digital economy, with the help of digital communications technology, has proven its strong vitality, even during the COVID-19 pandemic, and has become an important part of regional economic development, providing a possible path to rural revitalization. Taking the county as the research unit, this paper discusses the current situation, spatial differentiation patterns, and influencing factors of digital rural development construction in the YRB. According to our results, digital rural development in this region has been slightly better than the overall national level, but the decomposition indicators have not warranted optimism for the development of the digital economy, which has lagged behind the overall national level. Therefore, we believe that digital rural development in the YRB should promote the construction of the digital rural by tapping the potential of the digital economy through infrastructure construction.

The YRB has achieved good results in digital infrastructure construction, including resulted related to access and the popularization of mobile Internet and the integration of the digital economy into the residents’ daily lives. However, obvious shortcomings exist in digital production and digital marketing. A Taobao village is a typical representative of a new type of village specializing in e-commerce in the current era of the digital economy. Wang et al. [16] pointed out that traditional agricultural areas in the North China Plain were high-intensity gathering centers of Taobao villages, and Heze City in Shandong Province is a typical representative. This province has developed digital economies in areas such as Mudan District, Caoxian County, and Shan County. In comparison, the digital economies in the central and western regions lag to a certain extent. Compared to the lower reaches, the industrial structures of the middle and upper reaches are obviously different. In the future, the integration of the advantages and characteristics of rural industries into the construction of the digital economy to drive rural development is a problem worthy of attention. In addition, the YRB has 28 pilot counties, the prospects for development are optimistic, and there is also a need to improve digital infrastructure so that it can play a better role in driving the development of surrounding counties.

### 4.2. Driving Factors of Digital Rural Development in the YRB

The results of this study can deepen our understanding of the influence mechanism of digital rural development. Prior studies have concluded that the development of the digital economy was mainly affected by the levels of economic development, the level of information infrastructure, market size, and government support. Our research also confirms the influences of these factors on the construction of the digital rural. The influencing factors have different explanatory power for the spatial pattern of digital rural development in the YRB and its different reaches. Government expenditure and traffic infrastructure are influencing factors with a high level of influence in the YRB. Government expenditure and traffic infrastructure are influencing factors with a high level of influence in the YRB. In the initial stage, the government can play a role in regulating the market and various actors, making the construction of digital rural development more stable. For lower reaches, it has expanded beyond the stage of government-driven development and is mainly driven by industry structure. Convenient traffic conditions have become a new location requirement for digital rural development. It can not only strengthen foreign economic ties, but it can also save the transaction time of e-commerce, and enhance shopping experience.

Compared with other factors, economic level factors have relatively low explanatory power for digital rural development. However, when economic development interacts with other factors, the degree of explanation shows significant improvement. This indicates that economically backward areas have the opportunity to promote regional economic development from breakthroughs in the digital rural and to receive positive feedback. Accordingly, it is urgent to adjust the regional development strategy to promote sustainable digital rural development in the YRB.

### 4.3. Limitations and Prospects

Although a great deal of research work was carried out in this paper, there are still some limitations. Firstly, limited by the comparability of the index of digital rural development, this study only studied the spatial distribution characteristics and their driving forces in 2020. Therefore, in future studies, there is a need to obtain newly and comparable statistical data that analyze the spatiotemporal evolution of digital rural development in the YRB, providing a scientific reference for policy making.

Secondly, digital rural development is a complex process that involves the government, residents, e-platforms, etc. It is affected by many factors, such as ICT, individual behavior, industrial base, and government policy. In this paper, the explanatory variables selected were selected from the perspective of the economic basis and development potential. Related studies have shown that ICT plays a vital role in the development of the digital economy, but indicators such as optical cable line density and the density of mobile phone base stations are difficult to acquire for now. In future studies, we can obtain a comprehensive understanding of the mechanisms of digital rural development by case study.

## 5. Conclusions

This study used the Theil index, Moran’s I index, and a geodetector model at the county level to explore the spatial differentiation characteristics and factors influencing digital rural development in the Yellow River Basin (YRB). We found that:

(1) At present, digital rural development in the YRB has developed rapidly and has achieved good results. The average value of the digital rural development index is higher than the national average level in the same period. However, the development of the digital economy in the counties of the YRB does not favor optimism because it still lags behind the national average level. The digital rural development in the different reaches showed the trend of lower reaches > middle reaches > upper reaches. The differences within the counties in the upper reaches and in the counties in general are the main reasons for the differences in the level of digital rural development.
(2) The digital rural development and sub-index in the YRB have obvious spatial agglomeration characteristics. The high-value areas are mainly distributed in Qingdao, Heze in the southwest of Shandong Province, and in the counties surrounding Zhengzhou City in Henan Province, thus forming multiple agglomeration centers. The spatial clustering differentiation of digital rural development in the YRB is also obvious, and a large range of cold spots have formed in the upper reaches. The spatial difference of the digital economy index is the most obvious.
(3) The spatial patterns of digital rural development are influenced by economic basis and development potential. The impact of government expenditure and traffic infrastructure plays a leading role in digital rural development in the YRB. For the upper reaches, the influencing factors are more diverse and show a more obvious hierarchical structure. For the middle reaches, education level and the government play a dominant role in the middle reaches. The influencing factors in the lower reaches are singular and are mainly influenced by the industrial structure and the level of economic development. The influences of the interactions of each driving factor on digital rural development show double-factor and nonlinear enhancement effects. Accordingly, a coordinated regional development strategy is the key to promoting the digital rural development.

## Figures and Tables

**Table 1:** Index system and the detection results of the influencing factors of digital rural development.

Detection Factor	Factors	Unit
Economic basis	The per capita GDP (X1)	%
	The proportion of the secondary and tertiary industries output value in GDP (X2)	yuan
	The retail sale of consumer goods per capita (X3)	yuan
	The density of railway and highway (X4)	km/m^2^
Development potential	The urbanization level (X5)	%
	The per capita disposable income of households (X6)	yuan
	The general public budget expenditure per capita (X7)	yuan
	Average educational attainment (X8)	year
	The proportion of working-age people (X9)	%

**Table 2:** Digital rural development level in China and YRB and their decomposition index.

	Digital Rural		Digital Infrastructure		Digital Economy		Digital Governance		Digital Life	
	China	YRB	China	YRB	China	YRB	China	YRB	China	YRB
Max	122.083	94.938	120.004	118.175	154.871	111.381	96.812	92.418	125.080	92.602
Min	19.996	19.996	10.770	10.770	4.379	20.257	0.088	0.088	3.866	19.121
Average	55.734	56.736	77.600	79.114	47.067	46.217	48.537	53.818	48.210	49.229
SD	14.055	12.502	16.766	17.073	18.214	16.131	18.469	16.725	17.368	13.082
CV	0.252	0.220	0.216	0.216	0.387	0.349	0.381	0.311	0.360	0.266

**Table 3:** Average value of digital rural in YRB according to different ways of grouping.

	Digital Rural	Digital Infrastructure	Digital Economy	Digital Governance	Digital Life
YRB	56.736	79.114	46.217	53.818	49.229
upper reaches	44.402	61.146	34.164	44.997	41.725
middle reaches	56.483	82.138	44.983	51.834	47.664
lower reaches	65.637	88.414	55.984	62.134	56.171
municipality or county-level city	62.430	86.878	54.617	55.200	49.465
general county	53.686	74.957	41.719	53.078	49.102
national expiremental site	57.144	78.356	47.198	53.334	50.751

**Table 4:** Contribution decomposition of digital rural index decomposed by Theil index.

Group division based on regions	Intra-group			Sum-up	Inter group
	Upper reaches	Middle reaches	Lower reaches		
	21.69%	21.41%	14.39%	57.49%	42.51%
Group division based on administrative levels	Intra-group		Sum-up	Inter group	
	county	municipality or county-level city			
	62.90%	26.48%	89.38%	10.62%	

**Table 5:** Estimation of global Moran’s I for digital rural index in YRB, 2020.

	Digital Rural	Digital Infrastructure	Digital Economy	Digital Governance	Digital Life
Moran’s I	0.677	0.745	0.484	0.4478	0.370
*Z* score	27.081	29.419	19.574	17.845	14.432
*p*-value	0.001	0.001	0.001	0.001	0.001

**Table 6:** Detection factor q for digital rural development in the YRB.

Detector Factors	Yellow River Basin		Upper Reaches		Middle Reaches		Lower Reaches	
	*P*_*D*,*U*_	Sig.	*P*_*D*,*U*_	Sig.	*P*_*D*,*U*_	Sig.	*P*_*D*,*U*_	Sig.
X1	0.081	0.000	0.050	0.000	0.063	0.000	0.126	0.007
X2	0.215	0.000	0.252	0.000	0.179	0.000	0.150	0.000
X3	0.281	0.000	0.177	0.061	0.200	0.274	0.038	0.357
X4	0.352	0.000	0.356	0.000	0.185	0.965	0.106	0.994
X5	0.148	0.000	0.148	0.017	0.260	0.000	0.089	0.301
X6	0.183	0.000	0.096	0.544	0.362	0.000	0.065	0.132
X7	0.507	0.000	0.374	0.000	0.399	0.000	0.111	0.330
X8	0.267	0.000	0.275	0.000	0.451	0.000	0.096	0.685
X9	0.048	0.006	0.149	0.047	0.148	0.096	0.006	0.785

**Table 7:** Interactive detection results of impact factors of digital rural development in the YRB.

q	X1	X2	X3	X4	X5	X6	X7	X8	X9
X1	0.081								
X2	0.315 *	0.215							
X3	0.356 **	0.407 **	0.281						
X4	0.425 **	0.461 **	0.497 **	0.352					
X5	0.244 *	0.368 **	0.353 **	0.473 **	0.155				
X6	0.261 **	0.351 *	0.347 **	0.464 **	0.283 **	0.180			
X7	0.565 **	0.601 **	0.612 **	0.579 **	0.594 **	0.577 **	0.507		
X8	0.330 **	0.376 **	0.424 **	0.499 **	0.352 **	0.335 **	0.623 **	0.267	
X9	0.235 *	0.360 *	0.410 *	0.417 *	0.529 *	0.391 *	0.570 *	0.529 *	0.048

Notes: ** and * indicate double factor enhancement and nonlinear enhancement, respectively.

## Data Availability

The index of digital rural county 2020 utilized in this paper are obtained through the Institute for New Rural Development, Peking University. http://www.ccap.pku.edu.cn/nrdi/xmycg/yjxm/363361.htm, (accessed on 8 July 2022).

## Appendix A

**Table A1:** National experimental sites for digital rural development in the YRB.

Province	Prefecture Level City	County-Level Administrative Districts
Shanxi	Linfen	Xi county, Hongtong county
	Datong	Yunzhou district
	Jincheng	Gaoping city
Inner Mongolia	Hohhot	Tuoketuo county
	Erdos	Etuokeqian banner
Shandong	Zibo	Gaoqing county
	Taian	Feicheng city
	Binzhou	Huimin county
	Yantai	Haiyang city
Henan	Sanmenxia	Lingbao city
	Hebi	Qibin county
	Nanyang	Xixia county
	Luohe	linying county
Shaanxi	Weinan	Dali county
	Yangling	Yangling district
	Shangluo	Zuoshui county
	Hanzhong	Foping county
Gansu	Jiuquan	Yumen city
	Zhangye	Gaotai county
	Lanzhou	Gaolan county
Qinghai	Hainan Zang A.P.	Guinan county
	Haidong	Huzhu Tu Autonomous county
	Guoluo Zang A.P.	Maduo county
	Xining	Huangyuan county
Ningxia	Wuzhong	Yanchi county, Litong district
	Shizuishan	Pingluo county

Notes: A.P. is an abbreviation for Autonomous Prefecture.

## References

[1] Roberts E., Anderson B.A., Farrington J. (2017). A review of the rural-digital policy agenda from a community resilience perspective. J. Rural. Stud..

[2] Philip L.J., Townsend L., Roberts E., Beel D. (2015). Editorial: The rural digital economy. Scot. Geogr. J..

[3] Young J. (2016). Polar bear management in a digital Arctic: Inuit perspectives across the Web. Canad. Geogr..

[4] European Commission (2017). Digital Tourism. Actions to Help Tourism Businesses Go Digital..

[5] Chen Y.F., Liu Y.S. (2017). Research on the Mode and Mechanism of Rural Land Consolidation.

[6] Chen Y.F., Liu Y., Li Y.R. (2019). Agricultural development status and industrial prosperity path under the background of rural revitalization in China. Geogr. Res..

[7] Liu Y.S., Li Y.H. (2017). Revitalize the world’s countryside. Nature.

[8] Wang D.G., Zhu Y.J., Zhao M.F., Lv Q.Y. (2020). Multi-dimensional hollowing characteristics of traditional villages and its influence mechanism based on the micro-scale: A case study of Dongcun Village in Suzhou, China. Land Use Pol..

[9] Yang R., Pan Y.X. (2021). Spatial patterns, formation mechanism and coping strategies of rural vulnerability in China at the county level. Acta Geogr. Sin..

[10] Tilson D., Lyytinen K., Sørensen C. (2010). Research commentary—digital infrastructures: The missing IS research agenda. Inf. Sys. Res..

[11] The State Council The General Office of the CPC Central Committee and The State Council Issued the Outline of the Strategy for Digital Rural Development. http://www.gov.cn/zhengce/2019-05/16/content_5392269.htm.

[12] Rijswijk K., Klerkx L., Bacco M., Brunori G. (2021). Digital transformation of agriculture and rural areas: A socio-cyber-physical system framework to support responsibilisation. J. Rural Stud..

[13] Wang S., Yu N., Fu R. (2021). Digital rural construction: Action mechanism, realistic challenge and implementation strategy. Reform.

[14] Li C.M., Zhou Y. (2021). Influence of Digital Inclusive Finance on Rural Consumption: Based on Spatial Econometric Model. Econ. Geogr..

[15] Peng J.Z., Tao X.H., Xu L. (2019). Geographical Agglomeration Characteristic and Temporal & Spatial Evolution Mechanism of Digital Poverty in China. Econ. Geogr..

[16] Wang M.J., Yan Z.H., Yu B., Zhuo R.R., Guo X.W. (2022). Spatial pattern and change of e-commerce specialized villages and influence factors based on the data of Taobao villages in China from 2015 to 2020. Prog. Geog..

[17] Aruleba K., Jere N. (2022). Exploring digital transforming challenges in rural areas of South Africa through a systematic review of empirical studies. Sci. Afr..

[18] Wang W., Xu H.Y., Liu Y.X. (2022). Platform ruralism: Digital platforms and the techno-spatial fix. Geoforum.

[19] Fu B.J., Wang S., Shen Y.J., Cheng C.X., Li Y., Feng X.M., Liu Y.X. (2021). Mechanisms of human-natural system coupling and optimization of the Yellow River Basin. Bull. Natl. Nat. Sci. Found. China.

[20] Liu C.G. (2020). Spatial evolution of specialized villages and influencing factors in the Yellow River Basin. Resou. Sci..

[21] Shi L.N., Wang Y.S. (2021). Evolution characteristics and driving factors of negative decoupled rural residential land and resident population in the Yellow River Basin. Land Use Pol..

[22] Miao C.H., Zhang B.F. (2021). Regulation strategy of Zoning-Gradation-Classification for high-quality development in the Yellow River Basin. Econ. Geogr..

[23] (2022). Digital Village Project of Institute of New Rural Development. Peking University. Index of Digital Rural County 2020. http://www.ccap.pku.edu.cn/nrdi/docs/2022-05/20220530144658673576.pdf.

[24] (2020). Digital Village Project of Institute of New Rural Development. Peking University. Index of Digital Rural County 2018. https://www.saas.pku.edu.cn/docs/2020-09/20200929171934282586.pdf.

[25] National Bureau of Statistics of China (2020). China County Statistical Yearbook.

[26] Office of Leading Group of the State Council for the Seventh National Population Census (2022). Tabulation on 2020 China Population Census by County.

[27] Sinha A., Balsalobre-Lorente D., Zfar M.W., Saleem M.M. (2022). Analyzing global inequality in access to energy: Developing policy framework by inequality decomposition. J. Environ. Manag..

[28] Li B., Zhang W.Z., Yu J.H. (2016). A Study on Total factor energy efficiency and its difference in Resource-based Cities in China with consideration of environmental constraints. J. Natu. Resou..

[29] Liu C.X., Zhang X.D., Wang T., Chen G.Z., Zhu K., Wang Q., Wang J. (2022). Detection of vegetation coverage changes in the Yellow River Basin from 2003 to 2020. Ecol. Indic..

[30] Xue D., Yue L., Ahmad F., Draz M.U., Chandio A.A., Ahmad M., Amin W. (2022). Empirical investigation of urban land use efficiency and influencing factors of the Yellow River basin Chinese cities. Land Use Pol..

[31] Anselin L. (1995). Local indicators of spatial association—LISA. Geogr. Anal..

[32] Peng W.B., Han D.C., Yin Y., Yang Y., Shi X.F., Kuang J.S. (2022). Spatial evolution and integrated development of digital economy in Beijing-Tianjin-Hebei region. Econ. Geogr..

[33] Chen Y.B., Yin G.W., Wang S.H. (2022). Influencing factors of e-commerce development level at county scale in Shandong Province. Econ. Geogr..

[34] Wang B.Y., Tian J.F., Cheng L.S., Hao F.L., Han H., Wang S.J. (2018). Spatial differentiation of digital economy and its influencing factors in China. Sci. Geogr. Sin..

[35] Zhu W.J., Chen J.J. (2022). The spatial analysis of digital economy and urban development: A case study in Hangzhou, China. Cities.

[36] Preda M., Dincă A.L., Taloș A.M., Mareci A., Surugiu C., Surugiu M.R. (2022). Questioning the potential for achieving active ageing in Bucharest. J. Settl. Spat. Plan..

[37] Wang J.F., Li X.H., Christakos G., Liao Y.L., Zhang T., Gu X., Zheng X.Y. (2010). Geographical Detectors-Based Health Risk Assessment and its Application in the Neural Tube Defects Study of the Heshun Region, China. Int. J. Geogr. Inf. Sci..

[38] Wang J.F., Xu C.D. (2017). Geodetector: Principle and prospective. Acta Geogr. Sin..

[39] Zhang X., Kasimu A., Liang H., Wei B., Aizizi Y. (2022). Spatial and Temporal Variation of Land Surface Temperature and Its Spatially Heterogeneous Response in the Urban Agglomeration on the Northern Slopes of the Tianshan Mountains, Northwest China. Int. J. Environ. Res. Public Health.

[40] Wang S., Sun P., Sun H., Liu Q., Liu S., Lu D. (2022). Spatiotemporal Variations of Carbon Emissions and Their Driving Factors in the Yellow River Basin. Int. J. Environ. Res. Public Health.

